# Kolmogorov GAM Networks Are All You Need!

**DOI:** 10.3390/e27060593

**Published:** 2025-05-31

**Authors:** Sarah Polson, Vadim Sokolov

**Affiliations:** 1 Oxford University, Oxford OX1 2JD, UK; sarahmpolson@gmail.com; 2 Department of Systems Engineering and Operations Research, George Mason University, Fairfax, VA 22030, USA

**Keywords:** Kolmogorov GAM networks, Kolmogorov Superposition Theorem, additive models, Kolmogorov–Arnold Network KAN, deep learning, Transformers, LLMs, GAM, machine learning, Köppen function

## Abstract

Kolmogorov GAM (K-GAM) networks have been shown to be an efficient architecture for both training and inference. They are additive models with embeddings that are independent of the target function of interest. They provide an alternative to Transformer architectures. They are the machine learning version of Kolmogorov’s superposition theorem (KST), which provides an efficient representation of multivariate functions. Such representations are useful in machine learning for encoding dictionaries (a.k.a. “look-up” tables). KST theory also provides a representation based on translates of the Köppen function. The goal of our paper is to interpret this representation in a machine learning context for applications in artificial intelligence (AI). Our architecture is equivalent to a topological embedding, which is independent of the function, together with an additive layer that uses a generalized additive model (GAM). This provides a class of learning procedures with far fewer parameters than current deep learning algorithms. Implementation can be parallelizable, which makes our algorithms computationally attractive. To illustrate our methodology, we use the iris data from statistical learning. We also show that our additive model with non-linear embedding provides an alternative to Transformer architectures, which, from a statistical viewpoint, are kernel smoothers. Additive KAN models, therefore, provide a natural alternative to Transformers. Finally, we conclude with directions for future research.

## 1. Introduction

The landscape of modern machine learning has been shaped by exponential growth in computational power, particularly through advances in GPU technology and frameworks like PyTorch. While Moore’s law continues to drive hardware improvements, and CUDA algorithms have revolutionized our ability to process vast amounts of internet data, we pose the following question: can we achieve superior performance through mathematically efficient representations of multivariate functions rather than raw computational power?

In this paper, we introduce Kolmogorov-generalized additive models (K-GAM), a novel neural network architecture whose additive structure enables simplified training and accelerated inference through a marked reduction in parameters compared to traditional approaches. K-GAMs leverage Kolmogorov’s superposition theorem by utilizing a composition of two key components, namely, a universal Köppen embedding (functioning as a space-filling curve), followed by a trainable outer function, g:[0,1]→[0,1]. Unlike the original iterative look-up table approach proposed by Köppen and Sprecher, which faces NP-hard computational challenges, we implement the outer function using a ReLU neural network that can be efficiently trained using standard optimization techniques.

A fundamental challenge in machine learning lies in effectively handling high-dimensional input–output relationships. This challenge manifests itself in two distinct but related tasks. First, one task is to construct a “look-up” table (dictionary) for the fast search and retrieval of input–output examples. This is an encoding and can be thought of as a data compression problem. Second, and perhaps more importantly, we must develop prediction rules that can generalize beyond these examples to handle arbitrary inputs.

More formally, we seek to find a good predictor function f(x) that maps an input *x* to its output prediction *y*. In practice, the input *x* is typically a high-dimensional vector:

$$y=f\left(x\right)\;\;\mathrm{where}\;\;x=\left(x_{1},\ldots ,x_{d}\right)$$

Given a training dataset ${\left(y_{i},x_{i}\right)}_{i=1}^{N}$ of example input–output pairs, our goal is to train a model, i.e., to find the function *f*. The key question is as follows: *how do we represent a multivariate function so as to obtain a desirable f?*

We demonstrate that Transformer architectures can be decomposed into two fundamental operations: a particular form of embedding followed by kernel smoothing. Hence, K-GAM models are natural competitors to Transformers. The significance of this connection becomes clear when we consider that Transformers have largely supplanted alternative architectures across a wide range of applications. Previous work in this direction—[1,2]—demonstrated the potential of Köppen-based approaches only for 2×2 image reconstruction tasks. Our analysis extends this framework to encompass A wider spectrum of machine learning problems.

The rest of this paper is outlined as follows. Section 2 discusses the Kolmogorov superposition theorem. Section 3 introduces Kolmogorov-generalized additive models (K-GAMs). Section 4 describes Transformer architectures as kernel smoothers. Section 5 provides an application of GAM-Kolmogorov embeddings to the iris dataset. Section 6 concludes with directions for future research.

## 2. Kolmogorov Superposition Theorem (KST)

Kolmogorov demonstrated that any real-valued continuous function f(x) where $x=(x_{1},\ldots ,x_{d})$ defined on $E^{d}$ can be represented as a convolution of two single-variable functions:

(1) $$f\left(x_{1},\ldots ,x_{d}\right)=\sum_{q=0}^{2d}g_{q}\left(\Psi_{q}\left(x_{1},\ldots ,x_{d}\right)\right)$$

where $g_{q}$ are continuous single-variable functions defined on the *d*-dimensional unit cube $E^{d}={\left[0,1\right]}^{d}$. Kolmogorov further showed that the $\phi_{q}$ functions can be decomposed into sums of single-variable functions:

(2) $$\Psi_{q}\left(x_{1},\ldots ,x_{d}\right)=\sum_{i=1}^{d}\psi_{q,i}\left(x_{i}\right)$$

This result is known as the Kolmogorov representation theorem [3] and is often written in the following form:

(3) $$f\left(x_{1},\ldots ,x_{d}\right)=\sum_{q=0}^{2d}g_{q}\left(\sum_{i=1}^{d}\psi_{q,i}\left(x_{i}\right)\right)$$

Kolmogorov originally defined the inner functions as $\Psi_{q}\left(x_{1},\ldots ,x_{d}\right)$, q=0,1,…,2d, using the following property $\psi_{q}\left(S_{q,k}\right)\cap \psi_{q^{\prime }}\left(S_{q,k^{\prime }}\right)=\emptyset ,\;k\neq k^{\prime },\;q,=0,1,\ldots ,2d$. Then, for any integer d≥2, let $S_{q,k}$ be a family of mutually disjoint *d*-dimensional cubes $S_{q,k}\cap S_{q,k^{\prime }}=\emptyset ,\;k\neq k^{\prime },\;k,k^{\prime }=1,2,\ldots ,q=0,1,\ldots ,2d$. The diameter of each cube approaches zero as k→∞, and every point of a unit cube ${\left[0,1\right]}^{d}$ belongs to at least d+1 cubes $S_{q,k}$ for each *k*. Unfortunately, this derivation is not constructive and does not provide a practical algorithm for constructing the inner functions $\phi_{q}$.

Our goal is to show that it applies directly to the problems of machine learning. Furthermore, we interpret the architecture as a statistical model that allows us to draw on uncertainty quantification and approximation bounds. To understand the power of this representation from a machine learning perspective, we can think of $\psi_{q,i}\left(x_{i}\right)$ as features. Note that the number of features is then 2d+1 for the *d*-dimensional input. This representation is also remarkable in the sense that $\psi_{q}\left(x\right)$ can be viewed as an embedding, which is *independent* of *f*. For details, see Section 3.

### 2.1. Kolmogorov–Arnold Networks

A significant development has been the emergence of Kolmogorov–Arnold networks (KANs). The key innovation of KANs is their use of learnable functions rather than weights on the network edges. This replaces traditional linear weights with univariate functions, typically parametrized by splines, enhancing both representational capacity and interpretability. Ref. [4] established the first practical connection between KST and neural networks by showing that any KAN can be constructed as a three-layer MLP. Polson and Sokolov proposed Bayesian KAN networks.

Recently, Refs. [5,6] considered KST in the form of a sum of functions, represented as a two-layer model:

(4) $$f\left(x\right)=f\left(x_{1},\ldots ,x_{d}\right)=\left(g\circ \Phi \right)\left(x\right)$$

More explicitly,

(5) $$f\left(x_{1},\ldots ,x_{d}\right)=\sum_{q=0}^{2d}\sum_{p=1}^{d}\psi_{q,p}\left(x_{p}\right)$$

Ref. [7] presented a significant theorem regarding the approximation of generalized bandlimited multivariate functions using deep ReLU networks. The key finding is that deep ReLU networks can approximate generalized bandlimited functions without suffering from the curse of dimensionality. The paper utilizes an extension of Carathéodory’s theorem to infinite-dimensional spaces, which is crucial for the approximation of bandlimited functions. As a result, this paper provides a theoretical foundation for the effectiveness of deep learning in high-dimensional problems, especially for a class of functions relevant to many scientific computing applications.

One can think of KAN networks as NNs in which functions $\psi_{q,p}$ are estimated at the nodes, rather than weights. This makes them very flexible. A natural choice involves the use of splines for $\psi_{q,p}$; Ref. [8] proposed the application of KSNs (Kolmogorov spline networks).

Another advantage is that they can be trained using fast, high-dimensional algorithms such as the Newton–Kaczmarz algorithm [9]. The authors showed that KAN is not only a superposition of functions but also a special case of a tree of discrete Urysohn operators:

(6) $$U\left(x_{1},\ldots ,x_{d}\right)=\sum_{p=1}^{d}g_{p}\left(x_{p}\right)$$

This insight leads to a fast, scalable algorithm that avoids backpropagation, is applicable to any GAM model, and uses a projection descent method with a Newton–Kaczmarz scheme.

Recent research has explored various functional classes for the outer functions, including Wan-KAN [10], SineKAN [11], and functional combinations [12]. Ref. [13] demonstrated that KAN networks can outperform traditional MLPs, while [14] proved their optimality in terms of approximation errors. Ref. [15] discussed the Bayesian interpretation of the Kolmogorov–Arnold representation. KAN networks are widely trained with gradient descent. Ref. [9] proposed a Newton–Kaczmarz scheme. Bayesian methods are also available [16] and offer the potential for fewer parameters and more efficient inference. There are also optimal posterior concentration results [17].

Early theoretical groundwork for adaptive learning in KANs was laid by [18], who introduced the concept of counter-propagation networks, where outer functions self-organize in response to input–output pairs (x,y). Theoretical understanding has continued to advance, with [19] showing that Kolmogorov networks with two hidden layers can precisely represent continuous, discontinuous, and unbounded multivariate functions, depending on the activation function choice in the second layer. Ref. [20] extended this to three-layer networks for discontinuous functions.

### 2.2. Inner and Outer Functions

The theorem has seen several refinements over time. Ref. [21] made a crucial advancement by proving that the inner functions could be Hölder continuous. Ref. [22] later strengthened this result, showing that these functions could be Lipschitz continuous, although this required modifications to both the outer and inner functions.

We focus on the Kolmogorov–Sprecher formulation, as modified by [23,24,25]. The first representation is as follows:

(7) $$f\left(x_{1},\ldots ,x_{d}\right)=\sum_{q=0}^{2d}g_{q}\left(\Psi_{q}\left(x\right)\right)\;\;\mathrm{where}\;\;\Psi_{q}\left(x\right)=\sum_{p=1}^{d}\alpha_{p}\psi \left(x_{p}+q\alpha \right)$$

We will call this the Kq-GAM model to indicate the fact that there are *q* univariate outer functions. Ref. [7] provided an iterative algorithm to find $g_{q}$ based on the original Köppen formulation.

The second (and most parsimonious) representation is one in which there is only one univariate outer function! This expresses any multivariate continuous function as follows:

(8) $$f\left(x_{1},\ldots ,x_{d}\right)=\sum_{q=0}^{2d}g\left(\Psi_{q}\left(x\right)\right)\;\;\mathrm{where}\;\;\Psi_{q}\left(x\right)=\sum_{p=1}^{d}\alpha_{p}\psi \left(x_{p}+qa\right)+\delta_{q}.$$

Ref. [25] defines the $\delta_{k}$ term.

We will refer to this as the K-GAM model in our ML implementation. Bryant discussed the choice of offset $\delta_{q}$. Note that the complexity reduction is that there is only one g:[0,1]→[0,1] outer function that depends on *f*. The caveat involves finding a computation algorithm to compute *g* in polynomial time.

Here, the inner functions $\psi_{q}$ are constructed as sums of translated versions of a single univariate function ψ, known as the Köppen function. The Köppen function is monotone, Hölder continuous with smoothness parameters, and has fractal-like properties. Recent work by [26] provides a modern construction of the Köppen function and establishes theoretical approximation results with ReLU networks. Ref. [27] extended these theoretical results and showed that a function with a smaller number of parameters can be used. The author used λ-adic representation, where

(9) $$f\left(x_{1},\ldots ,x_{d}\right)=g\left(\sum_{p=1}^{d}\lambda^{-p}\psi \left(x_{p}\right)\right).$$

The structure of $\psi_{q}$ differs fundamentally from the approaches used in traditional neural networks, which rely on hyperplanes (as in ReLU networks) or decision trees. In $\psi_{q}$, a slice in any given univariate direction cuts the regions defined by the embedding in half.

The inner functions, $\psi_{q}$, partition the input space into distinct regions, and the outer function, *g*, must be constructed to provide the correct output values across the regions that the inner function defines. The outer function, *g*, can be determined via a computationally intensive process of averaging. For each input configuration, the inner functions, $\psi_{q}$, generate a unique encoding, and *g* must map this encoding to the appropriate value of f(x). This creates a dictionary-like structure that associates each region with its corresponding output value. Köppen made significant contributions by correcting Sprecher’s original proof of this construction process, with improvements to the computational algorithm later suggested by [28,29]. Braun further enhanced the understanding by providing precise definitions of the shift parameters $\delta_{k}$ and characterizing the topological structure induced by $\psi_{q}$.

A fundamental trade-off in KST exists between function smoothness and dimensionality. The inner functions, $\psi_{p,q}$, can be chosen from two different function spaces, with each offering distinct advantages. The first option is to use functions from $C^{1}\left(\left[0,1\right]\right)$, but this limits the network’s ability to handle higher dimensions effectively. The second option is to relax the smoothness requirement to Hölder-continuous functions ($\psi_{p,q}\in {\mathrm{Holder}}_{\beta }\left(\left[0,1\right]\right)$), which satisfy the inequality ${\left|\psi \left(x\right)-\psi \left(y\right)|<|x-y\right|}^{\beta }$. These functions are less smooth, but this “roughness” enables better approximation in higher dimensions.

### 2.3. Ridge and Projection Pursuit Regression

To understand the significance of this trade-off, we consider ridge functions, which represent a fundamental building block in multivariate analysis. Since our ultimate goal is to model arbitrary multivariate functions *f*, we need a way to reduce dimensionality while preserving the ability to capture nonlinear relationships. Ridge functions accomplish this by representing one of the simplest forms of nonlinear multivariate functions, requiring only a single linear projection and a univariate nonlinear transformation. Formally, a ridge function $f:R^{d}\to R$ takes the form $f\left(x\right)=g\left(w^{T}x\right)$, where *g* is a univariate function and $x,w\in R^{d}$. The non-zero vector *w* is called the direction. The term “ridge” reflects a key geometric property, that is, the function remains constant along any direction orthogonal to *w*. Specifically, for any direction *u*, such that $w^{T}u=0$, we have the following:

(10) $$f\left(x+u\right)=g\left(w^{T}\left(x+u\right)\right)=g\left(w^{T}x\right)=f\left(x\right)$$

This structural simplicity makes ridge functions particularly useful as building blocks for high-dimensional approximation. Ref. [30] provides bounds on the approximation error when a function with a finite spectral norm is approximated by ridge functions.

Ridge functions play a central role in high-dimensional statistical analysis. For example, projection pursuit regression approximates input–output relations using a linear combination of ridge functions [31,32,33]:

(11) $$f\left(x\right)=\sum_{q=0}^{2d}g_{q}\left(w_{q}^{T}x\right),$$

where both the directions, $w_{i}$, and functions, $g_{i}$, are variables, and $w_{i}^{T}x$ denotes one-dimensional projections of the input vector. The vector $w_{i}^{T}x$ is a projection of the input vector *x* onto a one-dimensional space, and $g_{i}\left(w_{i}^{T}x\right)$ denotes a feature calculated from the data. Ref. [34] used nonlinear functions of linear combinations, laying important groundwork for deep learning.

## 3. Kolmogorov-Generalized Additive Models (K-GAMs)

We take a different approach. Rather than using learnable functions as network node activations, we directly use the KST representation. This is a two-layer network with a non-differentiable inner function. The network’s architecture can be expressed as follows:

(12) $$f\left(x_{1},\ldots ,x_{d}\right)=\sum_{q=0}^{2d}g_{q}\left(z_{q}\right)$$

where the inner layer performs an embedding from ${\left[0,1\right]}^{d}$ to $R^{2d+1}$ via the following;

(13) $$z_{q}=\eta_{q}\left(x_{1},\ldots ,x_{d}\right)=\sum_{p=1}^{d}\lambda_{p}\psi \left(x_{p}+qa\right)$$

Here, $\lambda_{p}=\sum_{r=1}^{\infty }\gamma^{-(p-1)\beta (r)}$ is a *p*-adic expansion with $\beta \left(r\right)=\left(d^{r}-1\right)/\left(d-1\right)$ and γ≥d+2, with $a={\left(\gamma \left(\gamma -1\right)\right)}^{-1}$.

### 3.1. Köppen Function

The Köppen function ψ straightforwardly computes and provides the basis for the inner architecture of the network. The function ψ is defined through a recursive limit:

(14) $$\psi \left(x\right)=\lim_{k\to \infty }\psi_{k}\left(\sum_{l=1}^{k}i_{l}\gamma^{-l}\right)$$

where each x∈[0,1] has the following representation:

(15) $$x=\sum_{l=1}^{\infty }i_{l}\gamma^{-l}=\lim_{k\to \infty }\left(\sum_{l=1}^{k}i_{l}\gamma^{-l}\right)$$

and $\psi_{k}$ is defined recursively as follows:

(16) $$\psi_{k}\left(d\right)=\left\{\begin{array}{cc} d, & d\in D_{1}\\ \psi_{k-1}\left(d-i_{k}\gamma^{-k}\right)+i_{k}\gamma^{-\beta_{d}\left(k\right)}, & d\in D_{k},k>1,i_{k}<\gamma -1\\ \frac{1}{2}\left(\psi_{k-1}\left(d-i_{k}\gamma^{-k}\right)+\psi_{k-1}\left(d+\gamma^{-k}\right)\right)+i_{k}\gamma^{-\beta_{d}\left(k\right)}, & d\in D_{k},k>1,i_{k}=\gamma -1 \end{array}\right.$$

Here, the sets $D_{k}$ are defined as follows. $D_{k}$ is a uniform grid, with step size $\gamma^{-k}$, defined by the following:

(17) $$D+k=\left\{i\gamma^{-k},0\leq i\leq \gamma^{k}-1\right\}\subset \left[0,1\right)$$

There are $\gamma^{k}$ different points $0\leq x\leq 1-\gamma^{-k}$ on each grid $D_{k}$ where each point is as follows: $x=\sum_{l=1}^{l}i_{l}\gamma^{-l},i\in \left\{0,1,\ldots ,\gamma -1\right\}$.

**Figure 1:**
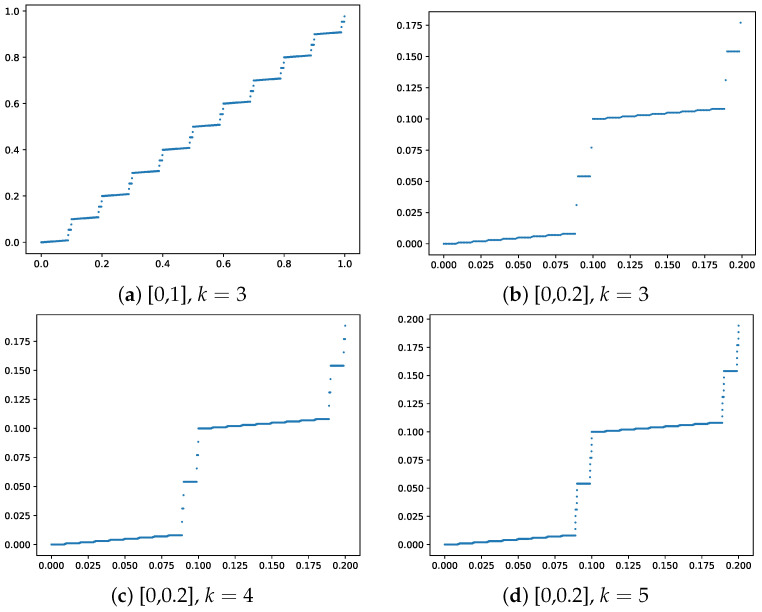
Köppen function $\psi_{k}$ for k=3,4,5, γ=10.

To illustrate, we show how the iterates update. Figure 1 shows the plot of the Köppen function $\psi_{k}$ for k=3 on intervals [0,1] and [0,0.2] (top row). The bottom row shows a “zoomed in” view of the function for k=4 and k=5 on the intervals [0,0.2]. The function has a fractal-like structure, with the number of discontinuities increasing with *k*. The Köppen function is a key component of the KST representation, providing a topological embedding of the input space that is independent of the target function *f*. This embedding is crucial for the network’s ability to capture complex multivariate relationships. Ref. [28] provided a detailed algorithm for computing the Köppen function and its properties. The author also provided a good, intuitive understanding of the function and its properties.

The most striking aspect of KST is that it leads to a generalized additive model (GAM) with fixed features that are independent of the target function *f*. These features, determined by the Köppen function, provide universal topological information about the input space, effectively implementing a k-nearest neighbors structure that is inherent to the representation. The outer function, *g*, is then responsible for learning the relationship between these features and the target function *f*. This separation of feature engineering and learning is a key advantage of K-GAM networks, enabling efficient training and inference.

**Theorem** **1** (K-GAM)**.** *Any function and dataset can be represented as a GAM with feature engineering (topological information) given by features $z_{k}$ in the hidden layer:*

(18) $$\begin{array}{cc} y^{\left(i\right)} & =\sum_{q=0}^{2d}g\left(z_{q}^{\left(i\right)}\right) \end{array}$$

(19) $$\begin{array}{cc} z_{q}^{\left(i\right)} & =\sum_{p=1}^{d}\lambda_{q}\psi \left(x_{p}^{\left(i\right)}+qa\right)+\delta_{q} \end{array}$$

*where ψ is a single activation function common to all nodes, known as the Köppen function, and g is a single outer function.*

**Proof.**  We can take the Köppen–Sprecher representation for y=f(x) and apply it *N* times to each output–input pair $\left(y^{\left(i\right)},x^{\left(i\right)}\right)$ and obtain a representation for $y^{\left(i\right)}=f\left(x^{\left(i\right)}\right)$. For example, for the K-GAM model and *g*-outer function, we have the following:

$$f\left(x_{1},\ldots ,x_{d}\right)=\sum_{q=0}^{2d}g\left(\sum_{p=1}^{d}\lambda_{q}\psi \left(x_{p}+qa\right)+\delta_{q}\right)$$

where $\lambda_{1}=1>\lambda_{2}\ldots >\lambda_{d}$ and a=1/(2d+1)(2d+2).

Now, for a dataset ${\left\{\left(y^{\left(i\right)},x^{\left(i\right)}\right)\right\}}_{i=1}^{N}$ where $y^{\left(i\right)}=f\left(x^{\left(i\right)}\right)$, we apply this representation to each data point:

$$\begin{array}{cc} y^{\left(i\right)} & =f(x_{1}^{\left(i\right)},\ldots ,x_{d}^{\left(i\right)})\\ \; & =\sum_{q=0}^{2d}g\left(\sum_{p=1}^{d}\lambda^{q}\phi \left(x_{p}^{\left(i\right)}+pa\right)+\delta_{q}\right)\\ \; & =\sum_{q=0}^{2d}g\left(z_{q}^{\left(i\right)}\right) \end{array}$$

where we define $z_{q}^{\left(i\right)}=\sum_{p=1}^{d}\lambda_{q}\phi \left(x_{p}^{\left(i\right)}+pa\right)+\delta_{q}$.

Ref. [7] proved that the outer function *g* can be approximated by a ReLU network to arbitrary precision. Therefore, we can replace *g* with a single ReLU network:

$$g\left(x\right)=\sum_{k=1}^{K}\beta_{k}\mathrm{ReLU}\left(w_{k}x+b_{k}\right),$$

where *K* is the number of neurons. We see that any function, *f*, and its corresponding dataset can be represented as a GAM with features engineered through the Köppen function ϕ.    □

Ref. [35] further developed this insight into a specialized two-layer ReLU network architecture. Specifically, the authors introduced a class of functions called Kolmogorov–Lipschitz continuous, where the outer function in the KST representation is Lipschitz continuous. Then they demonstrated that KL continuous functions can be approximated using a ReLU neural network with two hidden layers, achieving a dimension-independent approximation rate, and they introduced LKB-splines, created by using linear B-splines to replace the outer function in the KST representation.

As in [36], we can decompose with Lipschitz continuous functions. Note, the number of outer functions grows with the input dimension, *d*, not with the dataset size. The outer function, *g*, is shared across all terms, making this a true GAM representation with universal feature engineering provided by the inner Köppen function ϕ.

In a similar vein to [4], we show how this functional representation can be used to define a statistical model for applications in machine learning tasks [37,38]. We propose the following model:

(20) $$\begin{array}{cc} f(x_{1},\ldots ,x_{d}) & =\sum_{q=0}^{2d}g\left(\sum_{p=1}^{d}\alpha_{p}\psi \left(x_{p}+q\alpha \right)+\delta_{q}\right) \end{array}$$

(21) $$\begin{array}{cc} g(x) & =\sum_{k=1}^{K}\beta_{k}\mathrm{ReLU}\left(w_{k}x+b_{k}\right) \end{array}$$

where *K* denotes the number of neurons in the outer function’s architecture. The parameters ${\left(\beta_{k},w_{k},b_{k}\right)}_{k=1}^{K}$ can be learned using $L^{2}$-minimization and SGD. This architecture employs only two univariate activation functions, that is, a learned ReLU network for the outer layer and the Köppen function for the inner layer, which can be computed independently of *f*.

#### Note

A crucial aspect of KST-based architectures is their relationship to p-adic expansions and embeddings. This connection, first explored by [39] and recently advanced by [40], builds upon the foundational work of [41] on cellular neural networks in the 1980s, which included cellular automata as special cases.

For any input *x*, its p-adic expansion can be written as follows:

(22) $$x=\sum_{l=1}^{\infty }i_{l}\gamma^{-l}=\lim_{k\to \infty }\left(\sum_{l=1}^{k}i_{l}\gamma^{-l}\right)$$

where γ serves as the base of the expansion and $i_{l}$ denotes the digits in this representation. This expansion provides a natural embedding of the input space into a higher-dimensional representation, resembling contemporary kernel embedding techniques in deep learning [15,37,38].

### 3.2. Inference

An important property of an algorithm is how many parameters exist in the evaluation of the network, the so-called inference problem. The K-GAM network is notable for its relatively sparse parameterization.

Consider the class that corresponds to kernel estimators of the following form:

$$f\left(x\right)=\int K\left(x,t\right)d\mu \left(t\right)\;\;\mathrm{with}\;\int d\mu \left(t\right)\leq K\;\mathrm{and}\;x=\left(x_{1},\ldots ,x_{d}\right)$$

We call $K\left(x,t\right)=K_{t}\left(x\right)$ an $L_{\infty }$-atom if $|K_{t}\left(x\right)|\leq 1$. For such estimators, there exists an M-term approximation of *f*:

$$f_{M}\left(x\right)=\sum_{j=1}^{M}a_{j}K\left(x,t_{j}\right)\;\;\mathrm{such}\;\mathrm{that}{\left|\left|f-f_{N}\right|\right|}_{\infty }\leq CM^{-\frac{1}{2}},$$

with *C* independent of dimension *d*. While general approximations suffer from the curse of dimensionality with error $O(M^{-r/d})$ [42], where r denotes isotropic smoothness, certain restrictions yield dimension-independent bounds. For instance, when ∇f is in the $L^{1}$-Fourier class, the error becomes $O(M^{-1/2})$ independent of *d* [43].

The class of functions that we consider can be represented as $L^{1}$-combinations of $L^{\infty }$-atoms. For $f_{M}$, we will use superpositions of functions. For example, with $t:=(x_{0},s)$ and Gaussian atoms $K\left(x,t\right)=\exp\left(-{\left|\left|x-x_{0}\right|\right|}^{2}/s^{2}\right)$, see [44]. Let $t=(x_{0},k)$, where *k* is an orthant indicator. Then the resultant approximation is also of order $O(M^{-1/2})$, independent of *d*.

This framework naturally extends to incorporate dimension-reduction techniques like partial least squares (PLS). Given the input–output pairs ${\left(x_{i},y_{i}\right)}_{i=1}^{N}$, we can construct models of the following form:

$$Y=g({\hat{B}}_{PLS}X)$$

where ${\hat{B}}_{PLS}$ provides dimension reduction and *g* is a learnable transformation.

A more general class is found by the superposition of $2^{d}$ functions, each monotone for a different orthant. Then, the condition ∫d|μ|(t)≤1 manifests as a derivative condition:

$$\frac{\partial^{d}f}{\partial x_{1}\ldots \partial x_{d}}\in L_{1}.$$

This characterizes the space of functions with bounded mixed derivatives, which naturally leads to β-Hölder-smooth functions. For such β-Hölder-continuous functions, the network typically requires $O(M^{\beta })$ parameters (see [45] for further results on additive models and approximation properties in high dimensions).

Kolmogorov spline networks, designed for differentiable functions with bounded derivatives, satisfy the stronger property:

$$\left|\left|f-f_{M}\right|\right|=O\left(M^{-1}\right)$$

with the number of parameters given by $O(M^{2/3})$. This compares favorably to both the $O(M^{-1/2})$ approximation rate and the $O(M^{2})$ parameter count of the general one-layer-hidden feed-forward networks [8].

Deep ReLU networks, using max(x,0) activation, create separating hyperplanes across *L* layers, unlike cylinder sets (a.k.a. trees), which are a special case. Ref. [46] showed that energy-norm-based sparse grids and Kolmogorov approximation schemes have bounds that are independent of *d*.

Ref. [47] showed that efficient approximation schemes can be developed by the use of superpositions. Every continuous function of several variables can be represented by the superposition of functions with only two variables. Ref. [20] considered discontinuous functions.

As Kolmogorov might have said—there are no true multivariate problems, only superpositions of univariate affine ones!

### 3.3. Kernel Smoothing: Interpolation

The theory of kernel methods was developed by Fredholm in the context of integral equations [48]. The idea is to represent a function as a linear combination of basis functions, which are called kernels.

$$f\left(x\right)=\int_{a}^{b}K\left(x,x^{\prime }\right)d\mu \left(x^{\prime }\right)dx^{\prime }\;\;\mathrm{where}\;\;x=\left(x_{1},\ldots ,x_{d}\right)$$

Here, the unknown function, f(x), is represented as a linear combination of kernels, $K(x,x^{\prime })$, with unknown coefficients, $\phi (x^{\prime })$. The kernels are known, and the coefficients are unknown. The coefficients are found by solving the integral equation. The first work in this area was done by Abel [49], who considered equations of the form above.

Nowadays, we call those equations Volterra integral equations of the first kind. Integral equations typically arise in inverse problems. Their significance extends beyond their historical origins, as kernel methods have become instrumental in addressing one of the fundamental challenges in modern mathematics, that is, the curse of dimensionality.

Bartlett [50,51] proposed the use of kernels to estimate the regression function. The idea is to estimate the regression function, f(x), at point *x* by averaging the values of the response variable, $y_{i}$, at points $x_{i}$ that are close to *x*. The kernel is used to define the weights.

The regression function is estimated as follows:

$$\hat{f}\left(x\right)=\sum_{i=1}^{d}y_{i}K\left(x,x_{i}\right)/\sum_{i=1}^{d}K\left(x,x_{i}\right),$$

where the kernel weights are normalized.

Both Nadaraya [50] and Watson [51] considered the symmetric kernel $K\left(x,x^{\prime }\right)=K(∥x^{\prime }-x{∥}_{2})$, where $\left|\right|\cdot {\left|\right|}_{2}$ is the Euclidean norm. The most popular kernel of that sort is the Gaussian kernel:

$$K\left(x,x^{\prime }\right)=\exp\left(-\frac{∥x-x^{\prime }{∥}_{2}^{2}}{2\sigma^{2}}\right).$$

Alternatively, the two-norm can be replaced by the inner product: $K\left(x,x^{\prime }\right)=\exp\left(x^{T}x^{\prime }/2\sigma^{2}\right)$.

Later, Ref. [52] proposed using kernels to estimate the density function. The idea is to estimate the density function f(x) at point *x* by averaging the values of the kernel $K(x,x_{i})$ at points $x_{i}$ that are close to *x*. This idea was applied in many contexts by statisticians [53,54], machine learners [55], and engineers [56]. K-GAM builds upon these foundations.

Kernel methods are supported by numerous generalization bounds, which often take the form of inequalities that describe the performance limits of kernel-based estimators. A particularly important example is the Bayes risk for *k*-nearest neighbors (*k*-NN), which can be expressed in a kernel framework as follows:

$$\hat{f}\left(x\right)=\sum_{i=1}^{N}w_{i}y_{i}\;\mathrm{where}\;w_{i}:=K\left(x_{i},x\right)/\sum_{i=1}^{N}K\left(x_{i},x\right)$$

*k*-NN classifiers have been proven to converge to an error rate that is bounded in relation to the Bayes error rate, with the exact relationship depending on the number of classes. For binary classification, the asymptotic error rate of *k*-NN is, at most, $2R^{*}\left(1-R^{*}\right)$, where $R^{*}$ is the Bayes error rate. This theoretical bound suggests potential for improvement in practice. Cover and Hart proved that interpolated k-NN schemes are consistent estimators, meaning that their performance improves with the increasing sample size.

### 3.4. Training Rates

Consider the non-parametric condition regression, $y_{i}=f\left(x_{i}\right)+\epsilon_{i}$ where $x_{i}=\left(x_{1i},\ldots ,x_{di}\right)$. We wish to estimate $f(x_{1},\ldots ,x_{d})$ where $x=\left(x_{1},\ldots ,x_{d}\right)\in {\left[0,1\right]}^{d}$. From a classical risk perspective, we define the following:

$$R\left(f,{\hat{f}}_{N}\right)=E_{X,Y}\left({∥f-{\hat{f}}_{N}∥}^{2}\right)$$

where ∥.∥ denotes the $L^{2}\left(P_{X}\right)$-norm.

Under standard assumptions, we have an optimal minimax rate $\inf_{\hat{f}}\sup_{f}R\left(f,{\hat{f}}_{N}\right)$ of $O_{p}\left(N^{-2\beta /(2\beta +d)}\right)$ for β-Hölder-smooth functions *f*. This rate depends on the dimension *d*, which can be problematic in high-dimensional settings. By restricting the class of functions, better rates can be obtained, including ones that do not depend on *d*. In this sense, we avoid the curse of dimensionality. Common approaches include considering the class of linear superpositions (a.k.a. ridge functions) and projection pursuit models.

Another asymptotic result comes from a posterior concentration property. Here, ${\hat{f}}_{N}$ is constructed as a regularized MAP (maximum a posteriori) estimator, which solves the optimization problem:

$${\hat{f}}_{N}=\arg\min_{{\hat{f}}_{N}}\frac{1}{N}\sum_{i=1}^{N}{\left(y_{i}-{\hat{f}}_{N}\left(x_{i}\right)\right)}^{2}+\phi \left({\hat{f}}_{N}\right)$$

where $\phi (\hat{f})$ is a regularization term. Under appropriate conditions, the ensuing posterior distribution Π(f|x,y) can be shown to concentrate around the true function at the minimax rate (up to a logN factor).

A key result in the deep learning literature provides convergence rates for deep neural networks. Given a training dataset of input–output pairs ${\left(x_{i},y_{i}\right)}_{i=1}^{N}$ from the model y=f(x)+ϵ, where *f* is a deep learner (i.e., superposition of functions)

$$f=g_{L}\circ \ldots g_{1}\circ g_{0}$$

where each $g_{i}$ is a $\beta_{i}$-smooth Hölder function with $d_{i}$ variables, we have the following condition:

$$|g_{i}\left(x\right)-g_{i}\left(y\right)|<{\left|x-y\right|}^{\beta }.$$

Then, the estimator has an optimal rate:

$$O\left(\max_{1\leq i\leq L}N^{-2\beta^{*}/\left(2\beta^{*}+d_{i}\right)}\right)\;\mathrm{where}\;\beta_{i}^{*}=\beta_{i}\prod_{l=i+1}^{L}\min\left(\beta_{l},1\right)$$

This result can be applied to various function classes, including generalized additive models of the following form:

$$f_{0}\left(x\right)=h\left(\sum_{p=1}^{d}f_{0,p}\left(x_{p}\right)\right)$$

where $g_{0}\left(z\right)=h\left(z\right)$, $g_{1}\left(x_{1},\ldots ,x_{d}\right)=\left(f_{01}\left(x_{1}\right),\ldots ,f_{0d}\left(x_{d}\right)\right)$ and $g_{2}\left(y_{1},\ldots ,y_{d}\right)=\sum_{i=1}^{d}y_{i}$. In this case, $d_{1}=d_{2}=1$, and assuming *h* is Lipschitz, we obtain an optimal rate of $O(N^{-1/3})$, which is independent of *d*.

Ref. [57] shows that deep ReLU networks also have an optimal rate of $O(N^{-1/3})$ for certain function classes. For 3-times differentiable (e.g., cubic B-splines ), Ref. [58] found a rate of $O\left(N^{-3/7}\right)=O\left(N^{-3/(2\times 3+1)}\right)$. Ref. [8] found a rate $O(N^{-1})$ for Kolmogorov spline networks.

Finally, it is worth noting the relationship between expected risk and empirical risk. The expected risk, *R*, is typically bounded by the empirical risk plus a term of order $1/\sqrt{N}$:

$$R\left(y,f^{★}\right)\leq \frac{1}{N}\sum_{i=1}^{N}R\left(y_{i},f^{★}\left(x_{i}\right)\right)+O\left(\frac{∥f∥}{\sqrt{N}}\right)$$

where $f^{★}$ is the minimizer of the expected risk. However, in the case of interpolation, where the model perfectly fits the training data, the empirical risk term becomes zero, leaving only the $O(1/\sqrt{N})$ term.

## 4. Transformers as Kernel Smoothing

The fundamental connection between K-GAMs and Transformers becomes clear when we decompose Transformer architectures into their core operations, that is, embedding and kernel smoothing. This decomposition reveals why K-GAMs serve as natural competitors to Transformers, as both architectures leverage these same fundamental principles, albeit through different mechanisms. In both architectures, the embedding phase transforms raw inputs into a higher-dimensional space, where relationships can be more easily captured, while the smoothing phase aggregates information across this embedded space to produce outputs.

Transformers have become a main building block for various natural language processing (NLP) tasks and have been extended to other domains as well due to their effectiveness. The Transformer architecture is primarily designed to handle sequential data, making it well-suited for tasks such as machine translation, language modeling, text generation, and more.

The Transformer approach to embedding begins by converting input tokens into vectors in a high-dimensional space, augmented with positional information to preserve sequential structure. K-GAMs, in contrast, employ the Köppen function to transform inputs into a universal embedding space. This difference in embedding strategy reflects a fundamental trade-off, that is, Transformers learn task-specific embeddings that can adapt to particular domains, while K-GAMs leverage a universal embedding that can theoretically capture any continuous function.

Next, we will discuss the role of kernel smoothing in Transformers. Ref. [59] first introduced kernel smoothing for sequence-to-sequence learning through what became known as the attention mechanism. This approach estimates the probability of the next word in the sequence using a so-called context vector $c_{i}$, which is a weighted average of the vectors from the input sequence $h_{j}$:

$$c_{i}=\sum_{j=1}^{n}\alpha_{ij}h_{j},$$

where $\alpha_{ij}$ denotes the weights. The weights are defined by a kernel function, specifically a normalized exponential (softmax):

$$\alpha_{ij}=\frac{\exp\left(e_{ij}\right)}{\sum_{k=1}^{n}\exp\left(e_{ik}\right)}.$$

When used for self-supervised learning, this attention mechanism is called self-attention. When a sequence is mapped to a matrix *M*, it is called multi-head attention.

This formulation mirrors the structure of traditional kernel smoothing methods, with the key innovation being that, rather than using a fixed similarity measure like Euclidean distance, the energy function $e_{ij}=a\left(s_{i-1},h_{j}\right)$ is learned through a neural network. This neural network adaptively measures the similarity between the last generated element of the output sequence $s_{i-1}$ and the *j*-th element of the input sequence $h_{j}$, allowing the model to learn context-dependent attention patterns.

Both architectures use these phases to reduce the dimensionality of the learning problem while preserving the ability to capture complex relationships. The key difference lies in how they implement these phases; Transformers learn both the embedding and smoothing operations, while K-GAMs use a fixed, universal embedding (Köppen function) followed by learned smoothing.

The main advantage of using smoothing techniques is that they are parallelizable. This is the key draw of Transformers. Current language models such as BERT, GPT, and T5 rely on the Transformer approach. Further, Transformers have also been applied to computer vision and other domains. Their ability to capture long-range dependencies and their scalability have made them powerful tools for a wide range of applications. See Ref. [60] for further details.

## 5. Application

### 5.1. Simulated Data

We also apply the K-GAM architecture to a simulated dataset to evaluate its performance on data with known structures and relationships. The dataset contains 100 observations generated from the following function:

$$\begin{array}{cc} \; & y=\mu (x)+\epsilon ,\;\epsilon ∼N(0,1)\\ \; & \mu \left(x\right)=10\sin\left(\pi x_{1}x_{2}\right)+20{\left(x_{3}-0.5\right)}^{2}+10x_{4}+5x_{5}. \end{array}$$

**Figure 2:**
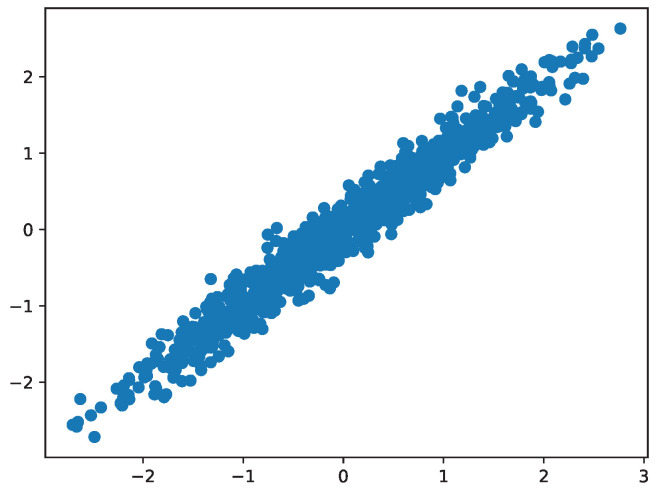
Scatter plot of the simulated dataset.

The goal is to predict the function y(x) based on the input *x*. The dataset is often used as a benchmark dataset for regression algorithms due to its diverse mix of relationships (linear, quadratic, nonlinear, Gaussian random noise) between the input features and the target function. The plot of μ (no noise) vs. *y* (noise) is shown in Figure 2.

We use the Köppen function to transform the five-dimensional input into a set of 11 features (2d+1). We then learn the outer function *g* using a ReLU network. To thoroughly investigate the model’s capabilities, we implement two distinct approaches to learning the outer function. The first approach uses different *g* functions for each feature, following the original KST formulation. This allows each function to specialize in capturing specific patterns, but it might be more difficult to train and has more parameters. The second approach uses a single *g* function for all features, as proposed by [61], providing a more unified and parameter-efficient representation.

**Figure 3:**
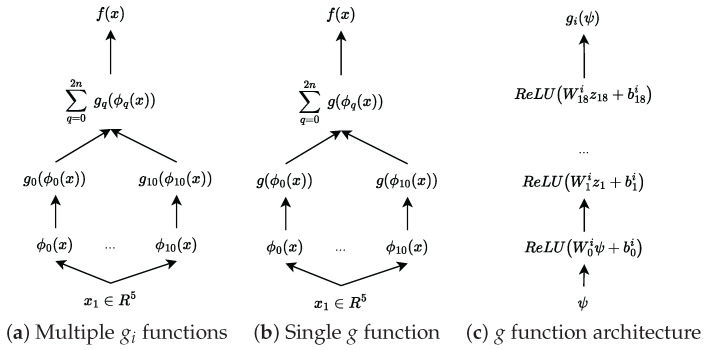
KST architecture for the simulated dataset.

**Figure 4:**
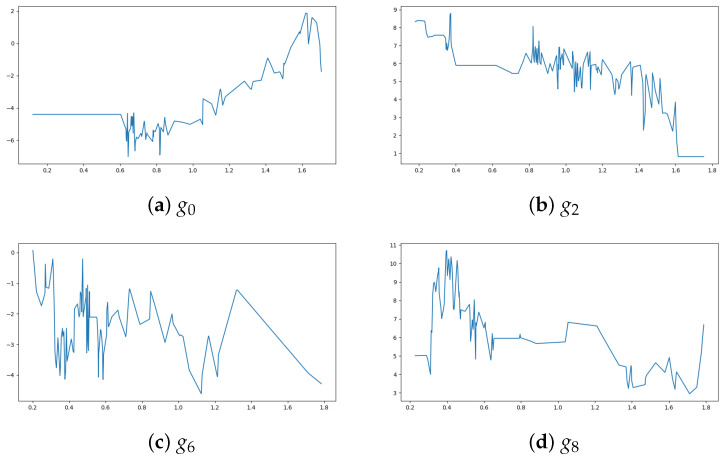
Examples of outer functions $g_{0},g_{2},g_{6},g_{8}$ for the simulated dataset.

Figure 3 illustrates these architectural choices in detail, showing how the information flows through each version of the model. For the first model with multiple $g_{i}$ functions, the dimensions of each $g_{i}$ are as follows: $W_{i}^{0}\in R^{16\times 1}$ and for j=1,…,18, $W_{i}^{j}\in R^{16\times 16}$. Exemplary outer functions are shown in Figure 4.

**Figure 5:**
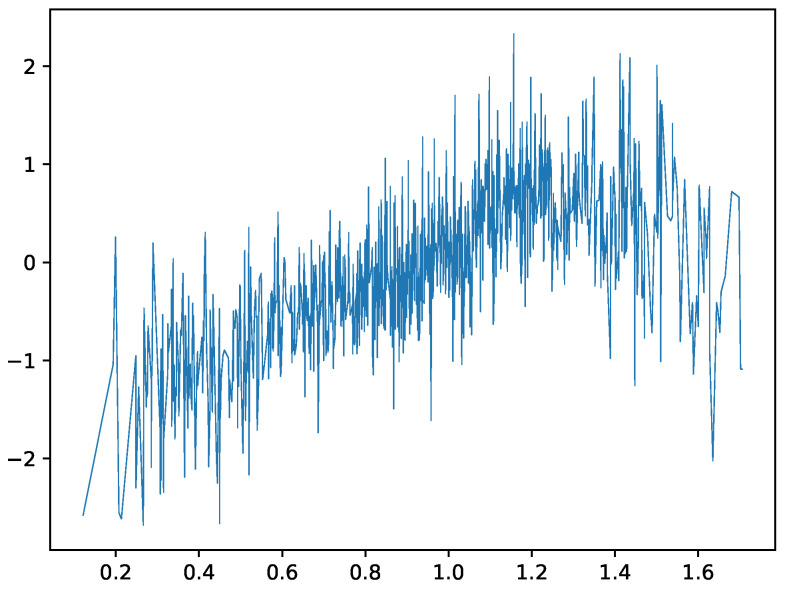
Plot of the single outer function, *g*, for the simulated dataset.

The next architecture, which uses only one function, *g*, for all features, maintains a similar structure to the multiple-*g* function approach. The only difference is in the dimensionality of the inner layers—we increase the width from 16 to 200. This increased capacity allows the single function to learn more complex patterns and compensate for the constraint of using just one function instead of multiple specialized ones. The behavior of this unified outer function is shown in Figure 5, where we can observe how it adapts to handle multiple feature transformations simultaneously.

### 5.2. Iris Data

**Figure 6:**
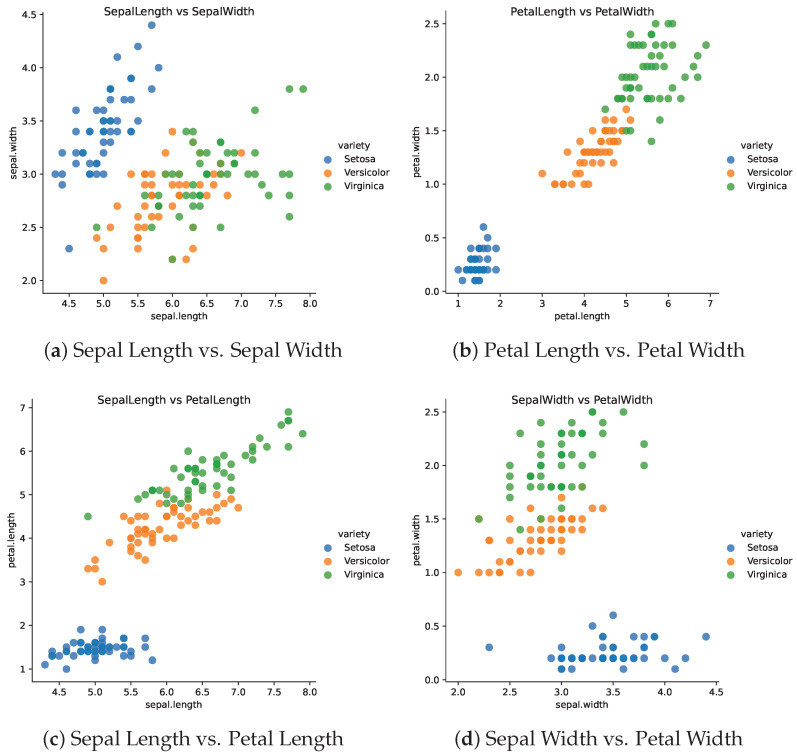
Scatter plots of the iris dataset.

We apply the KST architecture to an iris dataset. The iris dataset is a classic dataset in machine learning and statistics. It contains 150 observations of iris flowers. Each observation contains four features, namely, sepal length, sepal width, petal length, and petal width. The goal is to predict the species of the iris flower based on these features. The dataset contains three classes of iris flowers, namely, setosa, versicolor, and virginica. The dataset is often used as a benchmark dataset for classification algorithms. The dataset has five variables, which include four characteristics of the iris flower and the species of the flower. Figure 6 shows the scatter plots of the iris dataset.

We calculate the mean $\mu_{\mathrm{SL}}$ of the sepal length column and use a binary variable $y_{i}=\mathrm{Sepal}\;{\mathrm{Length}}_{i}>\mu_{\mathrm{SL}}$ as the output. We use the other three flower characteristics, $x_{1},x_{2},x_{3}$, as input variables. We use a classical GAM model to fit the data and compare it to KST-GAN. The classical generalized additive model is given by the following:

$$\mathrm{logit}\left(P\left(y=1|x\right)\right)=\beta_{0}+f_{1}\left(x_{2}\right)+f_{2}\left(x_{3}\right)+f_{3}\left(x_{4}\right),$$

where $f_{1},f_{2},f_{3}$ are smooth functions of the input features. The KST-GAN model is given by the following:

$$\mathrm{logit}\left(P\left(y=1|\psi \right)\right)=\beta_{0}+f_{1}\left(\psi_{1}\right)+\ldots +f_{7}\left(\psi_{7}\right),$$

where $\psi_{q}$ is the Köppen transformation function (in Appendix A) of the input features

$$\phi_{q}\left(x_{1},x_{2},x_{3}\right)=\sum_{i=1}^{3}\alpha_{i}\psi \left(x_{i}+aq\right),q=1,\ldots ,7.$$

We use m=2d+1=7 and k=6 to transform the input features into a set of seven features. We then learn the outer function *g* using a classical GAN approach. We use the mgcv package in R to fit the GAM model. This package uses a penalized likelihood approach to fit the model [62].

Table 1 compares the performances of the GAM and KST-GAM models. We also include a classical logistic regression model for comparison. The KST-GAM model has a higher AIC and BIC compared to the GAM model. The KST-GAM model has a comparable RMSE.

Table 2 and Table 3 show the confusion matrices for the GAM and KST GAM models, respectively. The KST GAM model has a lower accuracy compared to the GAM model.

**Figure 7:**
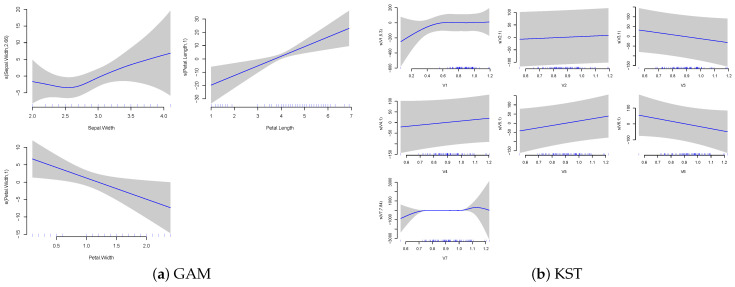
Generalized additive model for the iris dataset versus KST.

Figure 7 shows the original features plotted against those fitted by the GAM function for both original inputs and the KST-transformed inputs.

Overall, the iris example demonstrates the inability of the GAM model to capture the complex relationships between the transformed features and the target variable.

The comparison between traditional GAM and K-GAM approaches in Figure 7 shows some notable differences. The GAM plots show slightly smoother relationships between individual features and the output. For instance, petal width and petal length show particularly strong linear relationships with the target variable. In comparison, the K-GAM architecture seems to capture different aspects of the feature relationships. Notably, while one of the features retains a largely linear relationship, the other two features encode more complicated relationships. This difference demonstrates the effect of the Köppen function’s mapping.

Next, the simulated data study explores how K-GAM handles data with known structures, as well as the differences between using multiple outer functions and a single shared outer function within the K-GAM framework. The comparison between multiple $g_{i}$ functions (Figure 4) and a single *g* function (Figure 5) demonstrates the architectural flexibility of the approach. The single *g* function variant shows a highly variable pattern across its input range, showing that the increased dimension compensates for the reduced flexibility of having a single function by developing more complex internal representations. The ability to choose between multiple specialized functions or a single shared function provides a useful degree of freedom in the model’s design.

### 5.3. Image Processing

**Figure 8:**
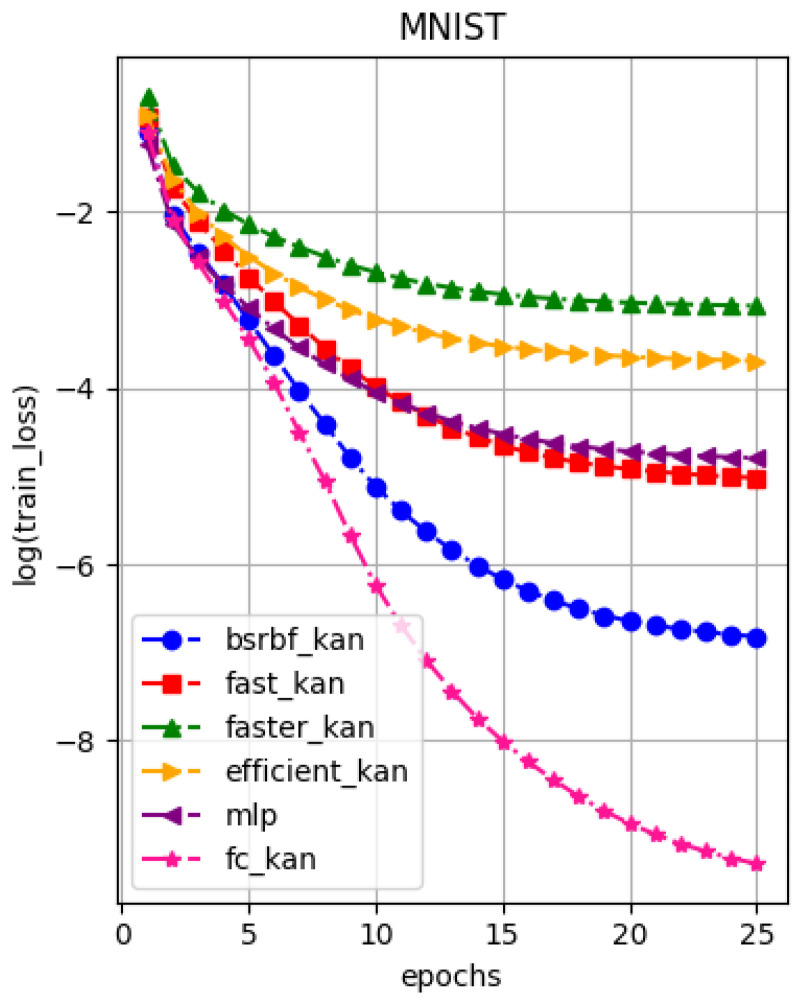
Functional KAN for the MNIST dataset.

A number of authors have proposed the use of Kolmogorov networks for image processing tasks (for example, see Refs. [1,2]). More recently, functional KANs were proposed in [12]. Figure 8 shows the error outputs for the MNIST dataset. Equivalent rates for the MNIST dataset can be found in [63].

Functional KAN methods have been shown to be effective for image processing tasks and have been shown to be promising in other tasks. Ref. [8] provided theoretical results for Kolmogorov networks applied to image segmentation tasks. Another alternative is deep partial least squares (DPLS) networks—a type of Kolmogorov network that uses partial least squares regression to learn the network weights [64].

## 6. Discussion

At its core, our work reinforces Kolmogorov’s profound insight, that is, there are no true multivariate problems, only superpositions of univariate affine ones. This principle guides our approach to efficiently decomposing complex multivariate functions into simpler univariate components through the Köppen transformation. The results demonstrate that while K-GAM can effectively model both real (iris) and simulated data, the internal representations it learns may be more complex than traditional GAM approaches. The approach appears particularly effective at capturing nonlinear patterns, although the interpretability of individual feature effects becomes more challenging due to the Köppen function transformation. Importantly, the K-GAM approach requires significantly fewer parameters compared to standard GAMs, as it leverages a shared embedding space through the Köppen function and can capture nonlinear relationships without requiring explicit interaction terms. However, most of the research since 2010 has been about increasing the depth of the networks and not about the width of the networks. The K-GAM approach is a step forward in the direction of increasing the width of the networks. The difference is that the Kolmogorov architecture is based on non-smooth functions, while the deep learning architecture is based on the composition of smooth functions. As shown by [65], it is impossible to obtain a universal approximation using a two-layer architecture with smooth functions.

Our findings suggest several promising directions for future research across both theoretical and practical domains. One priority involves the enhancement of the scalability of KST-based approaches, along with the characterization of the function classes for which K-GAM performs optimally over existing alternatives. Given K-GAM’s parameter efficiency, exploring Bayesian learning methods could provide principled approaches to uncertainty quantification and model regularization. An important optimization for K-GAM would be a specialized optimization algorithm capable of handling the discontinuities inherent in the Köppen function and the development of an efficient training algorithm specifically designed for high-dimensional problems.

These findings suggest that K-GAM networks represent a promising direction for efficient function approximation, particularly in scenarios where traditional deep learning approaches may be computationally intractable or parameter-inefficient. The combination of theoretical guarantees from Kolmogorov’s superposition theorem with modern machine learning techniques opens new avenues for both theoretical research and practical applications.

## Figures and Tables

**Table 1:** Summary of the classical GAM, KST GAM, and GLM models applied to the iris dataset.

	Classical GAM	KST GAM	GLM
(Intercept)	−1.758	18.064	−28.339
	(1.435)	(54.091)	(8.371)
Sepal.Width			2.669
			(1.499)
Petal.Length			6.377
			(2.090)
Petal.Width			−5.105
			(2.292)
Num.Obs.	105	105	105
R2	0.790	0.591	
AIC	43.0	207.3	50.6
BIC	59.3	258.1	61.2
Log.Lik.			−21.281
RMSE	0.22	0.29	0.26

**Table 2:** Confusion out-of-sample matrix for the GAM model applied to the iris dataset.

	Predicted 0	Predicted 1
Actual 0	21	1
Actual 1	2	21

**Table 3:** Confusion out-of-sample matrix for the KST GAM model applied to the iris dataset.

	Predicted 0	Predicted 1
Actual 0	19	3
Actual 1	8	15

## Data Availability

No new data were created or analyzed in this study.

## Appendix A. Function for Calculating the Köppen Transformation

def koppenFeatures(x,k):

qa = a*np.arange(m)

thf = koppenPhiv(np.add.outer(qa,x),k,gamma)

p = np.arange(1,n)

lmb = np.power(gamma,-np.multiply.outer(p,beta)).sum(axis=1)

lmb = np.insert(lmb,0,1)

thf = (thf@lmb).transpose()

return thf

def betaf(r):

  """

  Return the beta scalar for index r, as defined in Koppen paper

  Args:

    r (int): Index

  """

  print(f’n inside beta funciton is n’)

  return (pow(n,r)-1)/(n-1)

def lmb(beta,p):

  return pow(gamma,(1-p)*beta).sum()

def lambda_p(p):

  if p==1:

    return 1

  else:

    eps = 1

    tol = 10e-8

    total = 0

    r=1

    while eps>tol:

      eps = pow(gamma,(1-p)*beta[r])

      r+=1

      total += eps

  return total

def extractDigits(d,k,gamma):

  arr_d = []

  d=d*pow(gamma,k)

  d = round(d)

  for i in range(k):

    arr_d.insert(0,d % gamma)

    d = d//gamma

  return arr_d

def koppenPhiDigits(d,gamma):

  k = len(d)

  if k==1:

    return d[0]/gamma

  elif d[-1] < (gamma-1):

    return koppenPhiDigits(d[:-1],gamma) + d[-1]*pow(gamma,-beta[k])

.  else:

    d_prev = d[:-1]

    d_next = copy.deepcopy(d)

    while len(d_next)>0 and d_next[-1]==gamma-1:

      d_next = d_next[:-1]

    if len(d_next)==0:

      d_next = [gamma]

    else:

      d_next[-1] +=1

    return 0.5*(koppenPhiDigits(d_prev,gamma) + koppenPhiDigits

(d_next,gamma) + (gamma-2)*pow(gamma,-beta[k]))

    # return 0.5*(koppenPhiDigits(d[:-1],gamma) + koppenPhiDigits

(d_next,gamma) + (gamma-2)*pow(gamma,-beta[k]))

def koppenPhi(dk,k, gamma):

  assert dk<2

  assert dk>=0, f’Got negative input dk:.2e’

  if dk>1:

    return koppenPhi(dk-1,k,gamma)+1

  if k==1:

    return dk

  else:

    d = extractDigits(dk,k,gamma)

  return koppenPhiDigits(d,gamma)

koppenPhiv = np.vectorize(koppenPhi)
